# P-703. REspiratory Syncytial VIrus Knowledge, Attitudes and Practices (RESVIKAP) survey among older adults aged ≥50 years, carers and physicians in Asia-Pacific

**DOI:** 10.1093/ofid/ofaf695.915

**Published:** 2026-01-11

**Authors:** Yufan Ho, Lawrence Phillip Vandervoort, Lutz Beckert, Chien-Hsien Huang, Daisuke Kurai, Hoe Nam Leong, Ji Yun Noh, John Siu Lun Tam, Grant Waterer, Sumitra Shantakumar, Nisa de Souza, Aruni Seneviratna

**Affiliations:** GSK, Real World Evidence & Health Outcomes, Singapore, Singapore, Singapore; Oracle Life Sciences, Singapore, Singapore, Not Applicable, Singapore; Department of Medicine, University of Otago, Christchurch, New Zealand, Christchurch, Canterbury, New Zealand; Division of Infectious Disease, Department of Internal Medicine, Shin Kong Wu Ho Su Memorial Hospital, Taipei, Taiwan and College of Medicine, Fu Jen Catholic University, Xinzhuang, Taiwan, Taipei City, Taipei, Taiwan; Department of Clinical Infectious Diseases, Kyorin University School of Medicine, Tokyo, Japan, Tokyo, Tokyo, Japan; Rophi Clinic, Mount Elizabeth Novena Hospital, Singapore, Singapore, Not Applicable, Singapore; Division of Infectious Diseases, Department of Internal Medicine, Korea University College of Medicine, Seoul, South Korea, Seoul, Seoul-t'ukpyolsi, Republic of Korea; Department of Applied Biology & Chemical Technology, The Hong Kong Polytechnic University, Hong Kong, Hong Kong, Not Applicable, Hong Kong; Respiratory Medicine, Australia Royal Perth Hospital Unit and Internal Medicine, UWA Medical School, The University of Western Australia, Perth, Western Australia, Australia, Perth, Western Australia, Australia; GSK, Real World Evidence & Health Outcomes, Singapore, Singapore, Singapore; GSK, Real World Evidence & Health Outcomes, Singapore, Singapore, Singapore; GSK, Real World Evidence & Health Outcomes, Singapore, Singapore, Singapore

## Abstract

**Background:**

Respiratory syncytial virus (RSV) is a leading cause of respiratory infections (RI) in older adults, but RSV awareness remains low among older adults and their carers in the Asia-Pacific (APAC) region. With the recent approval of RSV vaccines for older adults, insights into RSV-related knowledge, attitudes, perceptions and practices (KAP) could inform targeted disease prevention strategies and policies.

This study assessed RSV-related KAP in older adults aged ≥50 years, their carers and physicians in APAC.

**Table 1.**
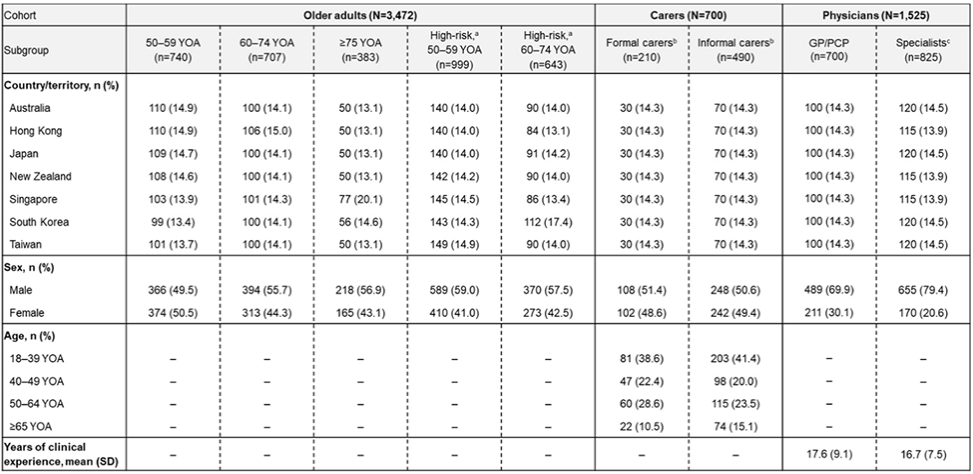
Sociodemographic characteristics of older adults ≥50 YOA, carers of older adults ≥50 YOA and physicians Cells marked with '–' represent data that were either not reported for or not applicable to a given cohort. [a] High-risk older adults included individuals with comorbidities such as respiratory conditions (e.g., chronic obstructive pulmonary disease, asthma), type I/II diabetes mellitus, cardiovascular disease/congestive heart failure, chronic kidney disease and liver disease. [b] Formal carers included professionals who provided nursing care, home care, rehabilitation/wellness or respite care services; informal carers included spouses, children or individuals who volunteered their care. [c] Specialists included pulmonologists/respiratory specialists (n=140), endocrinologists (n=140), cardiologists (n=140), gastroenterologists/hepatologists (n=140), nephrologists (n=140), geriatricians (n=63), infectious disease specialists (n=55) and family/community medicine specialists (n=7). GP: general practitioners; PCP: primary care physicians; SD: standard deviation; YOA: years of age.

**Figure 1.**
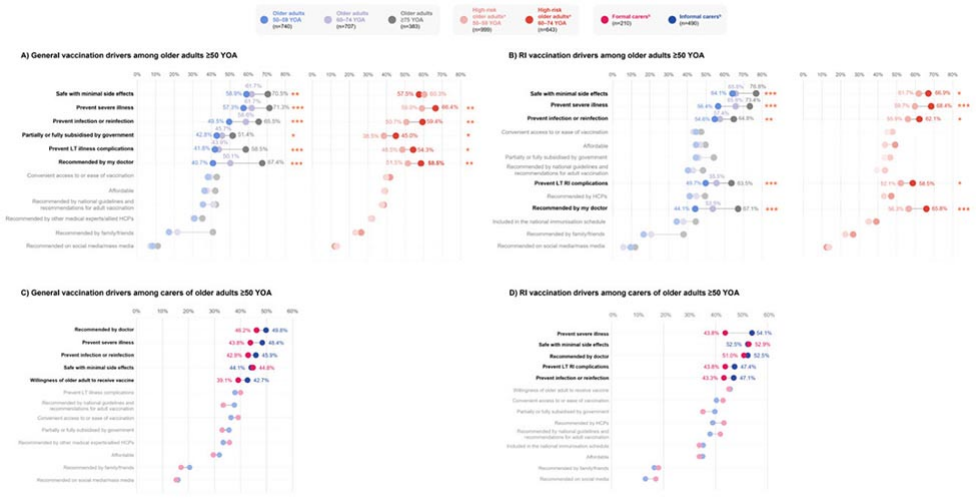
Key general and RI vaccination drivers among older adults ≥50 YOA and carers of older adults ≥50 YOA Percentage values represent the proportion of respondents for a subgroup within each cohort. Significant covariates (p<0.05) are reported for the differences between subgroups in a cohort: *p<0.05; **p<0.01; ***p<0.001. [a] High-risk older adults included individuals with comorbidities such as respiratory conditions (e.g., chronic obstructive pulmonary disease, asthma), type I/II diabetes mellitus, cardiovascular disease/congestive heart failure, chronic kidney disease and liver disease. [b] Formal carers included professionals who provided nursing care, home care, rehabilitation/wellness or respite care services; informal carers included spouses, children or individuals who volunteered their care. HCP: healthcare practitioner; LT: long-term; RI: respiratory infection; YOA: years of age.

**Methods:**

A cross-sectional study was conducted in Australia, Hong Kong, Japan, New Zealand, Singapore, South Korea and Taiwan. Qualitative interviews were conducted to refine survey tools, followed by quantitative surveys to explore the KAP focused on general vaccines and RSV prevention.

**Figure 2.**
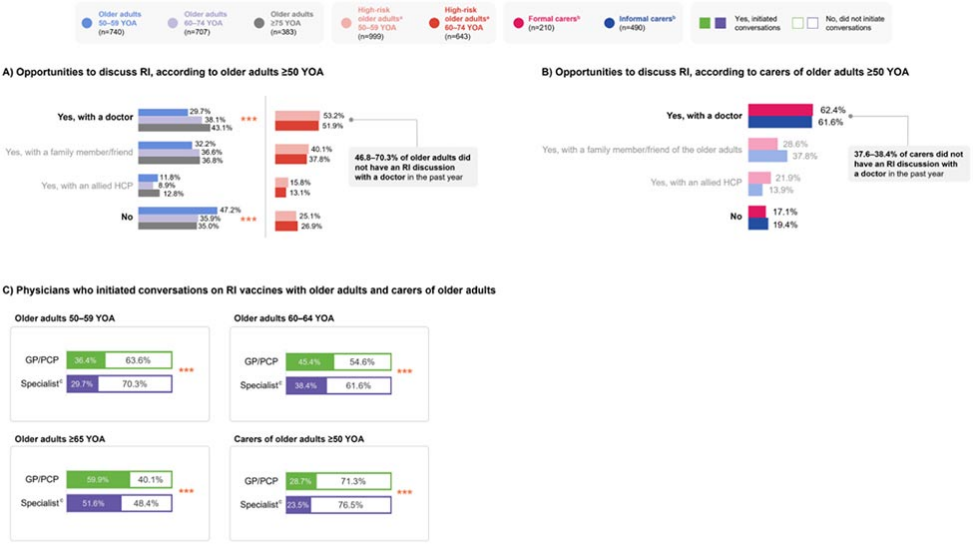
Opportunities for conversations between older adults ≥50 YOA/carers and physicians on RI or RI vaccines in the year prior to the date of the survey Percentage values represent the proportion of respondents for a subgroup within each cohort. Significant covariates (p<0.05) are reported for the differences between subgroups in a cohort: *p<0.05; **p<0.01; ***p<0.001. [a] High-risk older adults included individuals with comorbidities such as respiratory conditions (e.g., chronic obstructive pulmonary disease, asthma), type I/II diabetes mellitus, cardiovascular disease/congestive heart failure, chronic kidney disease and liver disease. [b] Formal carers included professionals who provided nursing care, home care, rehabilitation/wellness or respite care services; informal carers included spouses, children or individuals who volunteered their care. [c] Specialists included pulmonologists/respiratory specialists (n=140), endocrinologists (n=140), cardiologists (n=140), gastroenterologists/hepatologists (n=140), nephrologists (n=140), geriatricians (n=63), infectious disease specialists (n=55) and family/community medicine specialists (n=7). GP: general practitioners; HCP: healthcare practitioner; PCP: primary care physicians; RI: respiratory infection; YOA: years of age.

**Figure 3.**
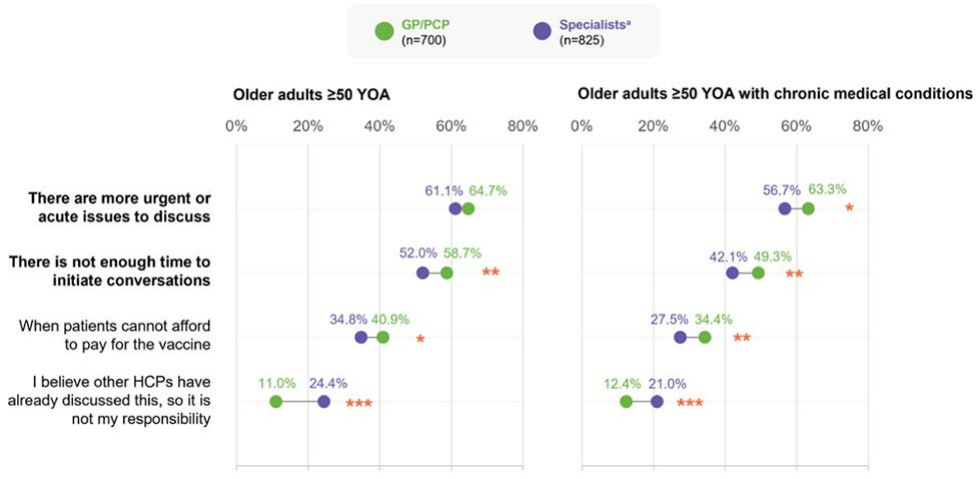
Reasons discouraging physicians from discussing RI vaccines with older adults ≥50 YOA and older adults ≥50 YOA with chronic medical conditions Percentage values represent the proportion of respondents for a subgroup within the physician cohort. Significant covariates (p<0.05) are reported for the differences between subgroups in a cohort: *p<0.05; **p<0.01; ***p<0.001. [a] Specialists included pulmonologists/respiratory specialists (n=140), endocrinologists (n=140), cardiologists (n=140), gastroenterologists/hepatologists (n=140), nephrologists (n=140), geriatricians (n=63), infectious disease specialists (n=55) and family/community medicine specialists (n=7). GP: general practitioners; HCP: healthcare practitioner; PCP: primary care physicians; RI: respiratory infection; YOA: years of age.

**Results:**

3,472 older adults (including 1,642 who were high-risk), 700 carers (formal/informal) and 1,525 physicians (specialists/generalists) were enrolled between April–July 2024 (Table 1).

Among older adults, key general and RI vaccination drivers included vaccine safety (57.5–70.5% and 61.7–76.8%, respectively), preventing severe illness (57.3–71.3% and 56.4–73.4%) and preventing (re)infection (49.5–65.5% and 54.6–64.8%; Fig. 1A–B). Among carers, key general and RI vaccination drivers included doctor’s recommendation (46.2–49.8% and 51.0–52.5%, respectively), preventing severe illness (43.8–48.4% and 43.8–54.1%), vaccine safety (44.1–44.8% and 52.5–52.9%) and preventing (re)infection (42.9–45.9% and 43.3–47.1%; Fig. 1C–D).

In the year prior to the survey, substantial proportions of older adults (46.8–70.3%) and carers (37.6–38.4%) had no opportunities to discuss RI with a doctor, while physicians initiated RI vaccine conversations in 23.5–59.9% of older adults/carers (Fig. 2). More urgent/acute issues (56.7–64.7%) and time constraints (42.1–58.7%) discouraged physicians from discussing RI vaccines with older adults (Fig. 3).

**Conclusion:**

This study identified vaccination drivers among older adults/carers (e.g., vaccine safety, efficacy). Limited opportunities for discussing RI vaccines with physicians were barriers to vaccination, highlighting unmet needs in prioritising vaccine conversations and preventive health, particularly for RSV.

Funding: GSK

**Disclosures:**

Yufan Ho, MSc, GSK: Employed by and hold financial equities in GSK Lutz Beckert, n/a, Asthma and Respiratory Society of New Zealand: Advisor/Consultant|AstraZeneca: Honoraria|GSK: Honoraria Daisuke Kurai, n/a, Alfresa Corporation: Honoraria|Asahi Kasei: Advisor/Consultant|Asahi Kasei: Grant/Research Support|Asahi Kasei: Honoraria|Beckman Coulter: Honoraria|Daiichi Sankyo: Advisor/Consultant|Gilead: Honoraria|GSK: Advisor/Consultant|GSK: Honoraria|GSK: Support for attending meetings and/or travel|Janssen: Advisor/Consultant|Janssen: Honoraria|Japanese Association for Infectious Diseases: Committee member involved in preparing the Clinical Practice Guide for RSV Infections for the Japanese Association for Infectious Diseases|KYORIN: Grant/Research Support|KYORIN: Honoraria|Kyowa Kirin: Honoraria|Maruishi Pharmaceutical: Grant/Research Support|MSD: Honoraria|Pfizer: Honoraria|Shionogi & Co.: Grant/Research Support|Shionogi & Co.: Honoraria|SRL, Inc.: Honoraria Ji Yun Noh, M.D.,Ph.D., GSK: Advisor/Consultant|GSK: Grant/Research Support|GSK: Honoraria John Siu Lun Tam, n/a, Asia-Pacific Alliance for the Control of Influenza: Director|The Chinese University of Hong Kong, Faculty of Medicine: Honoraria|The Chinese University of Hong Kong, School of Public Health: Honoraria Grant Waterer, n/a, GSK: Honoraria|Moderna: Honoraria|Pfizer: Honoraria Sumitra Shantakumar, n/a, GSK: Employed by and hold financial equities in GSK Nisa de Souza, n/a, GSK: Employed by GSK at the time of this study Aruni Seneviratna, n/a, GSK: Employed by and hold financial equities in GSK

